# Structural basis of AimP signaling molecule recognition by AimR in Spbeta group of bacteriophages

**DOI:** 10.1007/s13238-018-0588-6

**Published:** 2018-11-12

**Authors:** Xiangkai Zhen, Huan Zhou, Wei Ding, Biao Zhou, Xiaolong Xu, Vanja Perčulija, Chun-Jung Chen, Ming-Xian Chang, Muhammad Iqbal Choudhary, Songying Ouyang

**Affiliations:** 1 The Key Laboratory of Innate Immune Biology of Fujian Province, Biomedical Research Center of South China, College of Life Sciences, Fujian Normal University, Fuzhou, 350117 China; 2 Provincial University Key Laboratory of Cellular Stress Response and Metabolic Regulation, College of Life Sciences, Fujian Normal University, Fuzhou, 350117 China; 3 Shanghai Institute of Applied Physics, Chinese Academy of Sciences, Shanghai, 201204 China; 4 CAS Key Laboratory of Soft Matter Physics, Institute of Physics, Chinese Academy of Sciences, Beijing, 100190 China; 5 National Laboratory of Biomacromolecules, Institute of Biophysics, Chinese Academy of Sciences, Beijing, 100101 China; 6 Institute of Biotechnology, National Cheng Kung University, Tainan, 701 China; 7 State Key Laboratory of Freshwater Ecology and Biotechnology, Institute of Hydrobiology, Chinese Academy of Sciences, Wuhan, 430072 China; 8 H.E.J. Research Institute of Chemistry, International Center for Chemical and Biological Sciences, University of Karachi, Karachi, 75270 Pakistan

**Dear Editor,**

Quorum sensing (QS) is a widespread phenomenon in bacteria which enables them to participate in cell-to-cell communication by producing and responding to small signal molecules, thus synchronously altering their behavior depending on population density (Singh et al., 2000; Miller and Bassler, 2001). Through QS, bacteria coordinate processes such as expression of virulence factors (Slamti and Lereclus, 2002), biofilm formation (Parashar et al., 2011), sporulation (Perego et al., 1996), conjugation (Kozlowicz et al., 2006), antibiotic synthesis (Miller and Bassler, 2001; Whiteley et al., 2017) etc.

In Gram-positive bacteria, QS is mainly controlled by a family of cytosolic peptide-sensing regulators known as RRNPP, which is named for its representative members, i.e., Rap, Rgg, NprR, PlcR and PrgX. The reported structures of RRNPP members (Parashar et al., 2011; Gallego del Sol and Marina, 2013; Parashar et al., 2013) show a two-domain structure with an N-terminal helix-turn-helix (HTH) motif DNA-binding domain (DBD), a C-terminal tetratricopeptide repeat (TPR) domain containing the elements for signal peptide binding and oligomerization, and a short linker helix connecting the two domains. Despite the conserved tertiary structure of these regulators, structural analyses reveal unexpected diversity in the mechanism of activation and molecular strategies that couple the peptide-induced allostery to gene expression (Do and Kumaraswami, 2016).

Remarkably, a recent report described the use of a QS system for regulation of entry into lytic or lysogenic cycle by the *Bacillus*-infecting temperate phages phi3T and SPbeta (Erez et al., 2017). This system, termed *arbitrium*, is reminiscent of RRNPP-mediated QS found in Gram-positive bacteria, and is the first known example of QS in bacteriophages. The *arbitrium* system comprises three phage genes: (1) *AimP*, encoding the peptide that is processed into a 6 aa signaling peptide extracellularly, (2) *AimR*, encoding the intracellular receptor of AimP that is predicted to be structurally similar to RRNPP regulators (Fig. 1A), and (3) *AimX*, encoding a putative long non-coding DNA that functions as the negative regulator of lysogeny (Erez et al., 2017). During the initial stages of infection, the bacteriophages express AimP and AimR. As a transcription factor, AimR dimer induces expression of *AimX*, which promotes lytic cycle. Concurrently, the levels of mature AimP rise in extracellular medium. The concentration of AimP will eventually reach the point that its uptake by newly infected bacteria will be high enough to bind AimR and abolish transcriptional activation of *AimX*, thereby shifting the phage into lysogenic cycle. In this manner, the bacteriophages of SPbeta group are able to coordinate their reproduction, with preference for lytic cycle when there is abundance of host cells, and preference for lysogenic cycle in case of a dwindling bacterial population. However, the structures of AimR and AimR-AimP complex are still unknown, which impedes deeper understanding of the molecular mechanism underlying the switch between lytic and lysogenic cycles mediated by the *arbitrium* system.

**Figure 1:**
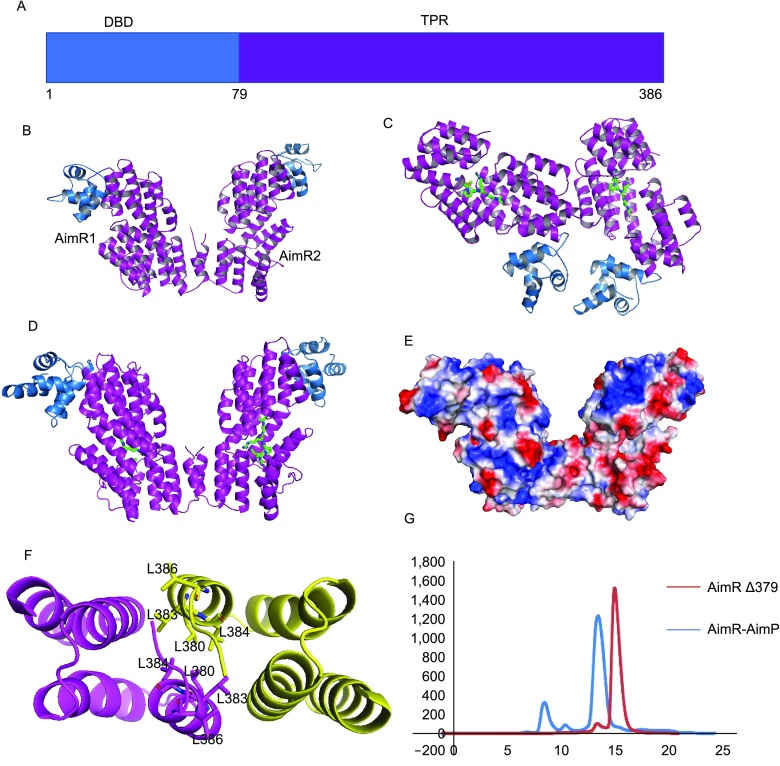
**Overall structures of apo AimR and AimR-AimP complex**. (A) Domain organization of AimR. (B) Structure of apo AimR. Two AimR molecules are found in one asymmetric cell unit. The AimR molecules are shown in cartoon, with colors depicting DBD and TPR domains as in Fig. 1A. (C) Structure of AimR-AimP complex in one symmetric cell unit. (D) The homodimer of AimR-AimP. AimRs are colored as in Fig. 1B, whereas AimP molecules are presented as yellow sticks. (E) Surface features of AimR homodimer by electrostatic potential (red, negative; white, neutral; blue, positive). (F) An overhead view of the leucine residues of C-terminal capping helix that form AimR dimerization interface. (G) Analytical size-exclusion chromatography of AimR_Δ379_ and wild-type AimR-AimP with Superdex 200 increase (GE Healthcare). Before the assay, AimP was incubated with AimR at 10:1 molar ratio for 30 min. Wild-type AimR-AimP was eluted at 13.5 mL (blue), whereas AimR_Δ379_ was eluted at 14.9 mL (red)

Here, we used X-ray crystallography to determine the structures of apo AimR (Fig. 1B) and AimR-AimP complex of *Bacillus subtilis* Spbeta group of bacteriophages (Fig. 1C and 1D). The crystals of apo AimR were obtained following extensive crystal screening and optimization assays. Due to the high flexibility of N-terminus, the electron density of the apo AimR DBD was weak, which made it challenging to build the structure of residues 1–55. After a strenuous effort, the structure of full-length apo AimR was solved at 2.63 Å resolution using single-wavelength anomalous diffraction method. The electron density of the N-terminus of AimR within the AimR-AimP complex was of better quality; using the structure of apo AimR as a model, the binary complex AimR-AimP was solved by molecular replacement and refined to 2.00 Å resolution. Detailed diffraction and refinement statistics are listed in Table S1.

The final structure of apo AimR contained two molecules in the asymmetric unit spanning residues 1–386 and a C-terminal His-tag (Fig. 1B). The two protomers within the asymmetric unit are nearly identical, with an average root mean square deviation (RMSD) of 0.159 Å (Fig. S1A). As predicted from its similarity with RRNPP proteins, the structure of AimR is characterized by two domains, i.e., the N-terminal HTH DNA-binding domain (residues 1–73) and the C-terminal TPR domain (residues 79–386), which are connected by a short linker (residues 74–78). A notable feature of AimR structure is that it comprises a total of 24 helical secondary structures, including 22 α-helices and two 3_10_-helices (Fig. S1C). While the first five helices form the DNA binding domain, the remaining α-helices are grouped into eight TPR motifs and a C-terminal capping helix. AimR and AimR-AimP are homodimers (Fig. 1E and 1F) and the dimerization is mediated by the C-terminal capping helix, with an approximate solvent-accessible surface of 810 Å^2^. The residues L380, L383, L384 and L386 contribute to dimerization by engaging in van der Waals interactions with matching residues from the other subunit (Fig. 1F). We tested an AimR deletion mutant lacking C-terminal capping helix (missing seven C-terminal amino acids, denoted AimR_Δ379_) by performing size-exclusion chromatography and found that dimerization was disrupted (Fig. 1G), thus confirming the importance of these residues in dimerization of AimR. Analysis of the surface electrostatic potential of AimR reveals a large positively charged area with potential DNA-binding activity located on the surface of DBD (Fig. S1A and S1B).

Although the overall structure of AimR shows high similarity to the classical RRNPP regulators, the sequence homology between AimR and the members of RRNPP is very low. However, a protein structure comparison on Dali server with AimR as a query retrieved 11 structures, among which RapJ, NprR, PlcR, RapF, RapI and RapH (all members of RRNPP family of proteins) had Z-scores higher than 13.

In the structure of AimR-AimP complex, two molecules of AimR are present in the asymmetric cell unit. One molecule of the AimP hexapeptide (GMPRGA) binds to one molecule AimR. The electron density of AimP was clearly visible within the complex (Fig. 2A). The TPR domain of AimR forms a deep concave pocket that accommodates a molecule of AimP, with a buried interface area of 758.6 Å^2^. The C-terminus alanine of AimP is facing the interior of the pocket, whereas the N-terminal glycine is located at the entry point (Fig. 2B and 2C). *In vitro* ITC assay demonstrates that full-length AimR specifically binds AimP with a *K*_d_ of 40 nmol/L (Fig. 2D). The interaction between AimR and AimP includes an extensive network of hydrogen bonds and hydrophobic contacts. The oxygen atoms from the peptide backbone of AimR form hydrogen bonds with four AimP residues: Q299_AimR_ and E300_AimR_ interact with the nitrogen moiety of G1_AimP_, N239_AimR_ forms hydrogen bonds with P3_AimP_, N202_AimR_ interacts with G5_AimP_, and R228_AimR_ contacts the oxygen moieties of A6_AimP_, whereas five hydrophobic residues from AimR (L205, L242, F276, F362 and L363) are involved in van der Waals interactions with the side chain of M2_AimP_.

**Figure 2:**
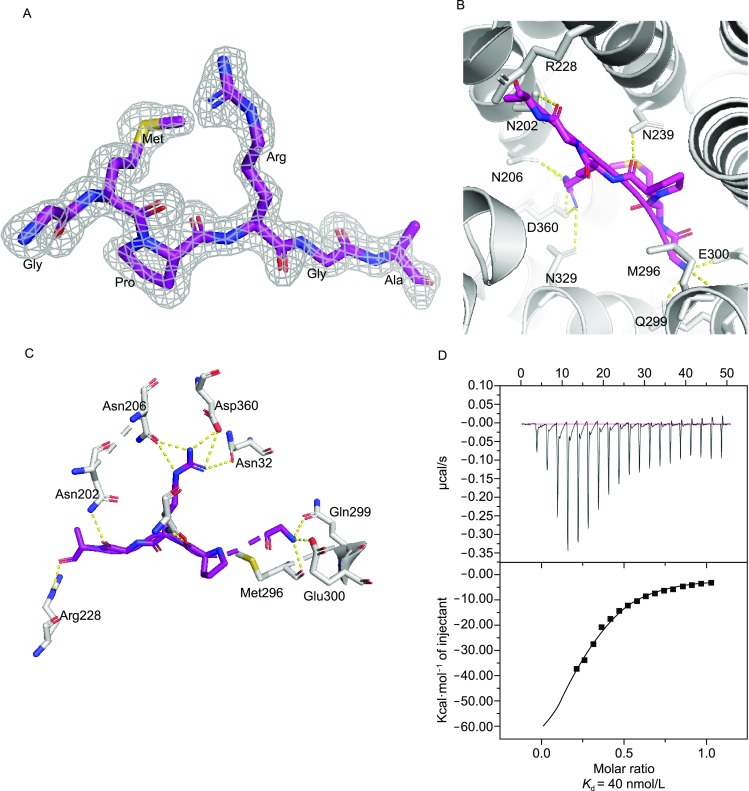
**Recognition of AimP hexapeptide (GMPRGA) by AimR from SPbeta phage**. (A) Electron density map of the AimP bound to AimR. The 2*F*o-*F*c map, calculated by simulated annealing without peptide in the structure, is shown contoured at 1.5 *σ* as a gray grid with the peptide in sticks colored by atom type. (B) The binding mode between AimR and AimP. AimR is shown as cartoon colored in white, whereas the residues that interact with AimP (N202, N206, N239, Q299, E300, N329 and D360) are shown as sticks colored by atom type. AimP is shown in stick representation with purple carbons. Hydrogen bonds are depicted as dotted yellow lines. (C) Similar to (B), but showing only interacting residues from another angle of view. (D) Binding affinity of wild-type AimR for AimP measured by ITC. The concentration of AimP used in assay was 200–500 μmol/L, and the concentration of AimR was 10–20 μmol/L

It is noteworthy to mention that AimR accommodates the GMRPGA peptide in a manner similar to the classical RRNPP transcription factors in bacteria. However, the mode by which AimP interacts with AimR differs from the previously studied interactions between RRNPP proteins and the corresponding signaling peptides. As opposed to the classical interaction between RRNPP and the signaling peptide in bacteria that does not include side chain-specific contacts, the guanidinium group of R4_AimP_ forms hydrogen bonds with three residues in AimR, namely N206, N329 and D360.

The importance of the AimR residues involved in AimP coordination was further confirmed by carrying out ITC assay with seven single point mutations (N202A, N206A, N239A, Q299A, E300A, N329A and D360A). While the value of dissociation constant for the wild-type AimR-AimP complex was 40 nmol/L, the abovementioned mutations reduced the AimP binding activity of AimR in the range between 0.14 μmol/L and 6.7 μmol/L (Fig. S2A–G). A sequence alignment of three AimRs from *Bacillus* phages revealed that all the residues except E300 are highly conserved (Fig. S1C). Thus, we conclude that the residues N202, N206, N239, N299, N329 and D360 of AimR play an essential role in binding AimP. Since previous research demonstrated that binding of AimP to AimR leads to silencing of the AimR-mediated *AimX* transcription and subsequent entry into lysogenic pathway (Erez et al., 2017), we further speculate that these residues form a switch that shifts phages between lytic and lysogenic cycle.

Active AimR dimer from the phi3T reportedly dissociates into inactive monomers upon AimP binding (Erez et al., 2017). However, our structure indicates that in the spBeta-derived AimR-AimP complex AimRs still exist as a dimer. This observation was corroborated by size-exclusion chromatography (GE healthcare, Superdex 200 increase) (Fig. 1F). Further analysis with the PISA program revealed that the AimR-AimP complex is most stable in heterotetramer conformation (i.e., an AimR dimer with each subunit binding one molecule of AimP), implying that in *arbitrium* system of spBETA phages AimP inactivates AimR through a mechanism distinct from the corresponding mechanism in phi3T phages.

While we were preparing our manuscript for submission, Wang et al. reported the structures of apo AimR (aa 43–386) and AimR_43-386_-AimP complex (Wang et al., 2018). Their structures share high similarity with the structures from present study (average RMSD of 0.223 Å for aligned apo AimR structures) (Fig. S3A). Moreover, similarly to Wang et al., we also noticed subtle conformational changes in N-terminal domain of AimR caused by binding of AimP. Prompted by this observation, we analyzed the intramolecular B-factor of AimR. The results revealed that the B-factor value of the N-terminal DBD, especially the first three helices, is notably higher than the rest of molecule, indicating that AimP binding may change the conformation of DBD (Fig. S3B–D), which in turn would reduce the DNA-binding ability of AimR. This is also in agreement with SAXS analysis indicating that AimP-bound AimR has an extended conformation when compared to AimP-free AimR (Wang et al., 2018).

Furthermore, our results are in line with another recent study, which found difference in infection dynamics between SPbeta phages and phi3T phages, and confirmed that SPbeta-derived AimR does not dissociate upon binding GMPRGA peptide (Dou et al., 2018). Altogether, these findings indicate that the molecular mechanism of AimR regulation does not only differ from bacterial RRNPP proteins but also from the more closely-related AimR in phi3T phages. However, future studies should investigate the structure of AimR bound to *AimX* gene in order to provide a clearer answer to how does AimP inhibit transcription of *AimX* by binding to AimR.

In summary, we reported the crystal structures of apo AimR and AimR-AimP complex from *arbitrium* system of SPbeta phage group. Our high resolution structures coupled with biochemical analyses shed new light on the molecular mechanisms underlying the switch between lysis and lysogeny in *arbitrium* QS system of SPbeta phages, and are in support of similar studies (Dou et al., 2018; Wang et al., 2018). The main findings in our study are outlined as follows: (1) AimR consists of 22 α-helices and two 3_10_-helices. The first three α helices in N-terminal DNA-binding domain of apo AimR show a high degree of flexibility. (2) The TPR domain of AimR forms a concave pocket in which a number of conserved residues bind the AimP hexapeptide GMPRGA with high affinity through an extensive network of hydrogen bonds and hydrophobic interactions. Although AimR accommodates the signaling peptide similarly to RRNPP proteins in bacteria, the nature of interactions between AimR and AimP is distinct. (3) The dimerization of AimR is mediated by the C-terminal capping helix. Unlike the AimR in phi3T phages, AimR dimer in SPbeta phages does not dissociate into monomers upon AimP binding, but instead appears to be further stabilized. This was inferred from observations in crystallization assays, the crystal structures of apo AimR and AimR-AimP complex, and computational analysis with PISA.

To conclude, we would like to propose an amended mechanistic model of *arbitrium* system in SPbeta phages, which takes into consideration all three relevant studies (Dou et al., 2018; Wang et al., 2018, and the present study): in the absence of AimP hexapeptide GMPRGA, AimR possesses a highly flexible N-terminal DNA-binding domain. When binding the *AimX* gene locus, the DBD domain adopts a conformation that allows specific interactions with DNA, which are primarily mediated by the first three α helices in DBD. However, AimP binding induces a second conformational change in AimR that is characterized by an extended but more rigid DBD, which distorts interactions between AimR and DNA, and results in decreased (rather than abolished) DNA binding. This, in turn, shifts the equilibrium between lysis and lysogeny in phage population towards lysogeny. Such model can more clearly explain not only the differences in infection dynamics between SPbeta phages and phi3T phages (Dou et al., 2018) but also the lack of obvious conformational variations between apo AimR and AimR-AimP complex (Wang et al., 2018 and the present study).

## FOOTNOTES

The atomic coordinates and structure factors have been deposited in the Protein Data Bank (PDB) under the accession codes 6IPX for apo AimR and 6IM4 for AimR-AimP complex.

We thank the staff at beamline BL-17U1 of Shanghai Synchrotron Radiation Facility for their help with X-ray diffraction data collection.

This work was supported by the Ministry of Science and Technology of China grants 2014CB910400 and the National Nature Science Foundation of China grants 31770948, 31570875, 31800159 and 81590761. We thank Fujian Normal University for financial support.

Xiangkai Zhen, Huan Zhou, Wei Ding, Biao Zhou, Xiaolong Xu, Vanja Perčulija, Chun-Jung Chen, Ming-Xian Chang, Muhammad Iqbal Choudhary and Songying Ouyang declare that they have no conflict of interests.

This article does not contain any studies with human or animal subjects performed by the authors.

## Electronic supplementary material

Below is the link to the electronic supplementary material.

Supplementary material 1 (DOCX 14401 kb)
