# Supplementary material for: Structural basis of AimP signaling molecule recognition by AimR in Spbeta group of bacteriophages
Source: Protein Cell. 2018 Nov 12;10(2):131–6. doi: 10.1007/s13238-018-0588-6 (PMC6340889; doi:10.1007/s13238-018-0588-6)
Supplement: Supplementary file 1 — Supplementary material 1 (DOCX 14401 kb) [file 13238_2018_588_MOESM1_ESM.docx]

**Materials and Methods**

**Protein expression and purification**

DNA sequence encoding full-length AimR from *Bacillus* *subtilis* SPbeta phage was cloned into the pET-28a vector with a C-terminal 6×His-tag. The construct was introduced into *E.coli* BL21 (DE3) cells. The recombinant strains were cultured in LB medium at 37°C until the OD_600_ reached 0.8. After 16 h of induction with 0.5 mM IPTG at 16°C, the cells were harvested by centrifugation at 5, 000 rpm for 30 min at 4°C. The pelleted cells were then resuspended in the lysis buffer containing 50 mM Tris-HCl (pH 8.0) and 150 mM NaCl, and subsequently lysed by ultrasonication. The lysate was then centrifuged at 17,000 rpm for 30 min at 4°C. Protein purification was carried out following the routine procedure (Jiang et al., 2017). Briefly, the supernatant was loaded onto a Ni-NTA column (Qiagen). The target protein was eluted with 50 mM Tris-HCl (pH 8.0) containing 150 mM NaCl and 250 mM imidazole. The fractions containing purified protein were pooled, concentrated to 0.5 ml and loaded onto a Superdex200 increase column (GE Healthcare) equilibrated with buffer containing 20 mM Tris-HCl (pH 8.0) and 150 mM NaCl. All the AimR mutant proteins were purified following the same protocol.

**Crystallization, data collection and structure determination**

Crystal screening of apo AimR and the AimR-AimP complex were performed simultaneously. The concentration of AimR used for crystallization was 15 mg/ml. In case of AimR-AimP complex, AimP was added at 5:1 molar ratio. Crystallization screening was performed using the hanging drop vapor diffusion method at 16°C. Crystallization drops contained 0.5 μl of the protein solution mixed with 0.5 μl of reservoir solution. Diffraction quality crystals of both apo AimR and AimR-AimP complex were obtained in 0.1 M HEPES (pH 6.5-7.5), 20% (v/v) PEG 4000, and 50 mM magnesium acetate. Crystals were harvested with 20% (v/v) ethylene glycol as a cryoprotectant before flash freezing them in liquid nitrogen.

The protein concentration of the selenomethionine derivate of AimR used for crystallization was ~20 mg/ml. Crystals of SeMet-labeled AimR were grown in the condition described above. 25% (v/v) ethylene glycol was used as a cryoprotectant when the crystals were harvested.

X-ray diffraction data were collected on beamline BL-17U1 at Shanghai Synchrotron Radiation Facility (SSRF). The diffraction images were processed with the HKL-2000 program (Otwinowski and Minor, 1997). Further data processing was performed using the CCP4 suite (Winn et al., 2011). The phase was identified using single anomalous diffraction from Se-Met by program AutoSol. Model building and crystallographic refinement were carried out in COOT (Emsley et al., 2010) and PHENIX (Adams et al., 2010). The interactions were analyzed with PyMol (http://www.pymol.org/) and PDBsum (Laskowski, 2007). The figures were generated in PyMol. Detailed data collection and refinement statistics are listed in Table S1.

**Site-directed mutagenesis**

The QuikChange Site-Directed Mutagenesis Kit (Stratagene) was used to generate AimR mutants. The designed mutants were verified by full-length sequencing.

**Isothermal titration calorimetry (ITC) assay**

ITC experiments were performed using a MicroCal iTC200 instrument (Malvern Instruments Ltd.). All the samples were prepared in a buffer containing 20 mM Tris-HCl (pH 8.0) and 150 mM NaCl. Except for the titrant or titrate, all other components of the solution were adjusted to the same levels before titration. Typically, AimP (200–500 μM) in a syringe was titrated into a sample cell containing AimR (10–20 μM) at 25°C following the multiple injections method. The resulting isotherms were recorded and fitted with Origin software (OriginLab).

**
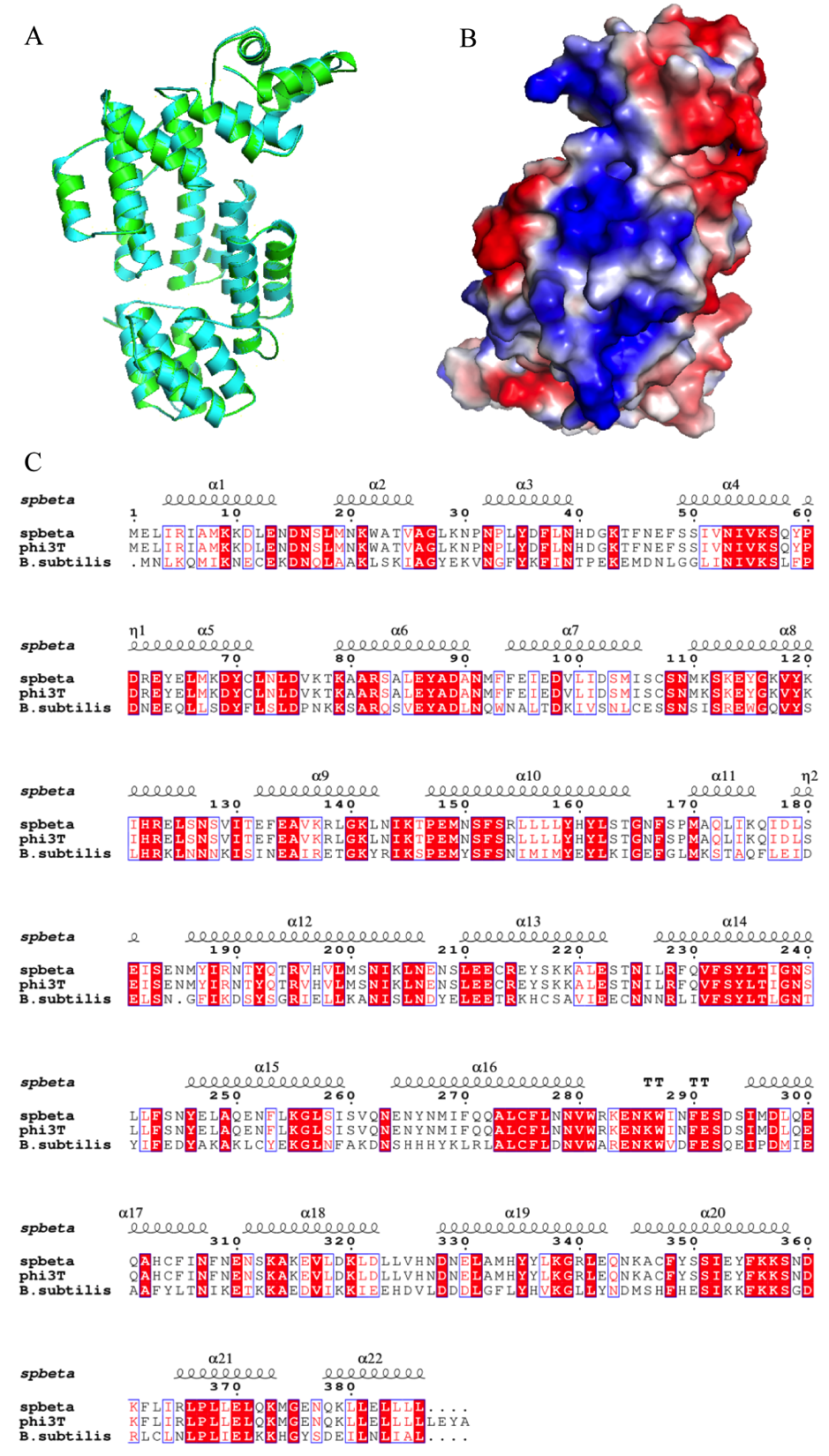
**

**Figure S1. Structural analysis of AimR.** (A) The two AimR protomers which form the asymmetric cell unit were superpositioned to show that they are identical to each other (root mean square deviation of 0.223 Å). (B) Electrostatic potential surface of AimR. The potential is represented by colors ranging from blue (positive charge) to red (negative charge). The positively charged patch with potential DNA-binding activity can be observed at the N-terminal end of the AimR DBD. (C) Sequence alignment between AimRs from SPbeta*,* phi3T and SPZ1 phages. Conserved residues are boxed in red with white letters. The alignment was generated by ESPript 3 (http://espript.ibcp.fr/ESPript/ESPript/).


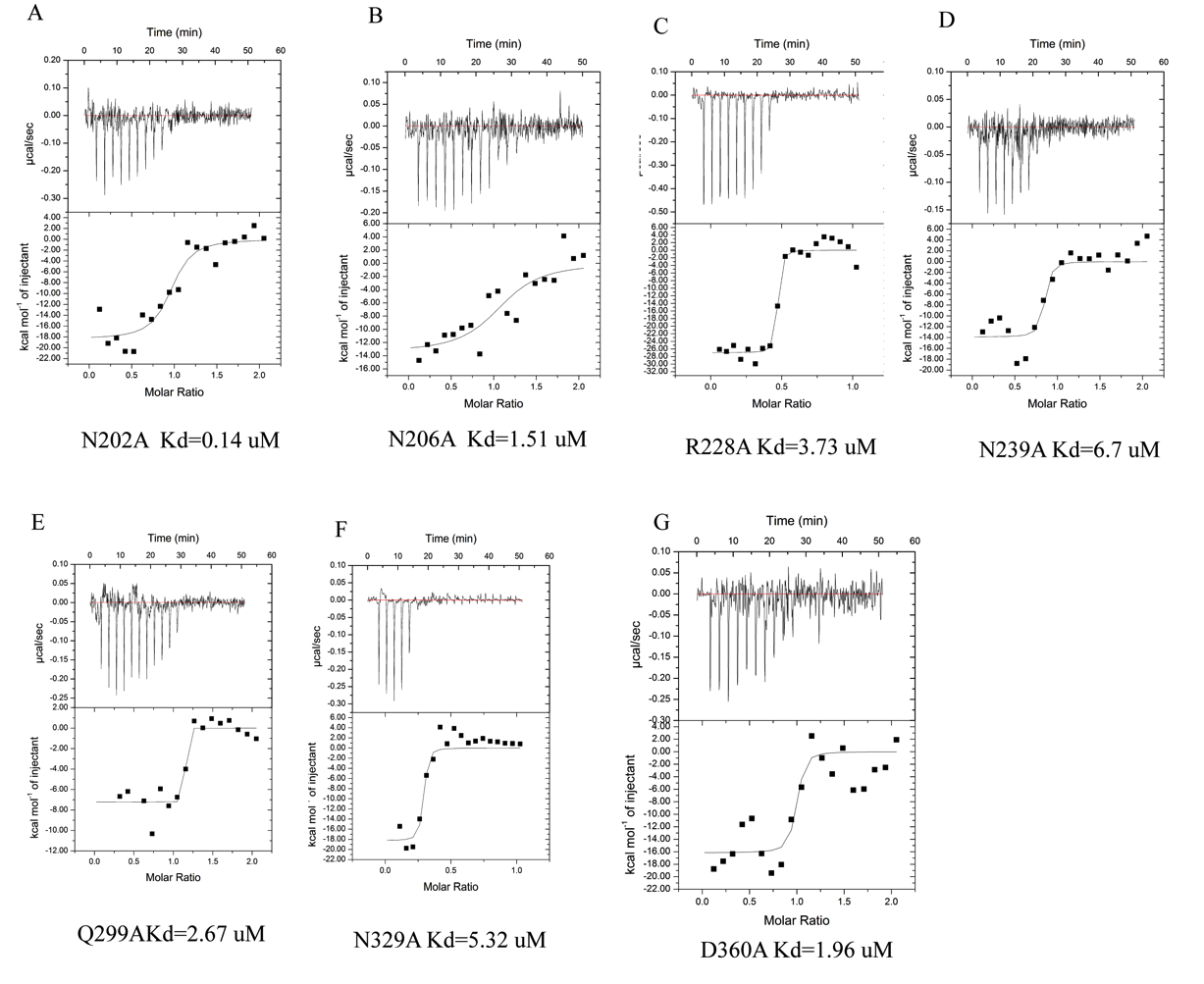


**Figure S2. Mutational analysis of seven amino acid residues from AimR involved in AimP binding with *in vitro* ITC.** AimP was titrated into each single-point mutant (A-G) to test binding affinities. Calculated dissociation constants are given below each panel, next to the respective description of single-point mutations. The concentration of AimP used for ITC was 200-500 μM, and the concentration of AimR was 10-20 μΜ.


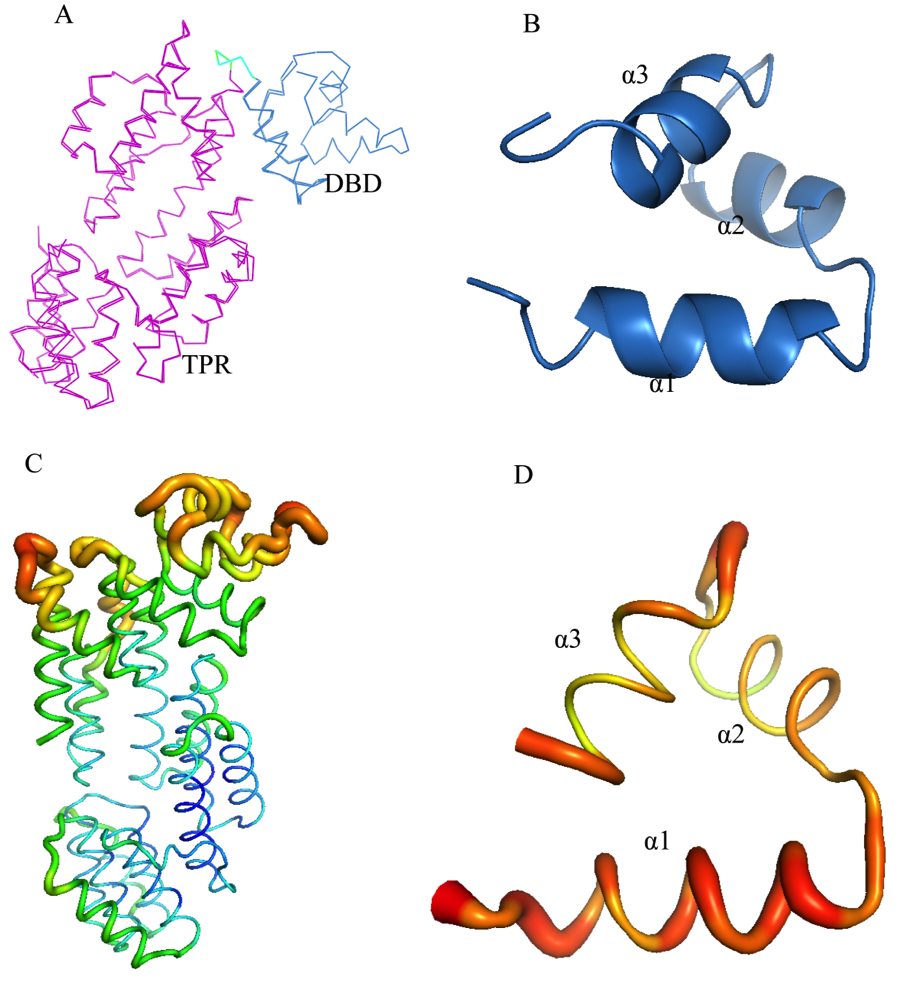


**Figure 3.** **Structural alignment of apo AimR from the present study with the recently reported AimR structure (Wang et al., 2018; PDB ID: 5Y24) and analysis of intramolecular B-factor values.** (A) Alignment of the AimR from the present study with the recently reported AimR structure indicates a high degree of similarity between the structures (average root mean square deviation of 0.223 Å). The structures are shown as ribbons, with N-terminal DBD are colored in blue, C-terminal TRP domain in purple, and the short linker in green. (B) A close-up view of the first three N-terminal helices of AimR. (C) Analysis of intramolecular B-factor values in AimR. The structure of AimR is shown in cartoon, with blue representing the lowest and red the highest B-factor values. The size of the tube also reflects the B-factor values: the higher the B-factor, the thicker the tube. (D) The N-terminus of AimR possesses highest B-factor values within AimR.

**Table S1. X-ray crystallography data collection and refinement statistics**

| **Dataset** | **SeMet-AimR** | **AimR** | **AimR-AimP** |
| --- | --- | --- | --- |
| **Data collection** |  |  |  |
| Wavelength (Å) | 0.9791 | 0.9791 | 0.9791 |
| Space group | *P*2_1_2_1_2 | *P*2_1_2_1_2 | *P*2_1_2_1_2 |
| Cell dimensions  a, b, c (Å) | 115.00, 220.80, 33.57 | 120.46, 214.71, 33.70 | 121.07, 213.59, 33.85 |
| α, β, γ (°) | 90.00, 90.00, 90.00 | 90.00, 90.00, 90.00 | 90.00, 90.00, 90.00 |
| Resolution (Å) | 2.30 | 2.63 | 2.00 |
| R_merge_ | 0.181 | 0.075 | 0.185 |
| CC1/2 | 0.995 | 0.997 | 0.982 |
| *I*/*σ*(*I*) | 16.5 | 2.0 | 18.0 |
| Completeness (%) | 99.45 | 98.9 | 99.83 |
|  |  |  |  |
| **Reﬁnement** |  |  |  |
| Resolution (Å) |  | 2.63 | 2.00 |
| No. reﬂections |  | 49620 | 60703 |
| Completeness (%) |  | 99.22 | 99.74 |
| R_work_ |  | 0.23 | 0.2211 |
| R_free_ |  | 0.28 | 0.2729 |
| No. atoms  Protein |  | 6415 | 7011 |
| Ramachandran plot (%) |  |  |  |
| Favored region |  | 95.99 | 98.34 |
| Allowed region |  | 3.75 | 1.36 |
| Outliers region |  | 0.26 | 0.00 |
| PDB No. |  | 6IPX | 6IM4 |

**References**

Adams, P.D., Afonine, P.V., Bunkoczi, G., Chen, V.B., Davis, I.W., Echols, N., Headd, J.J., Hung, L.W., Kapral, G.J., Grosse-Kunstleve, R.W., et al. (2010). PHENIX: a comprehensive Python-based system for macromolecular structure solution. Acta Crystallogr D Biol Crystallogr 66, 213-221.

Emsley, P., Lohkamp, B., Scott, W.G., and Cowtan, K. (2010). Features and development of Coot. Acta Crystallogr D Biol Crystallogr 66, 486-501.

Jiang, Y., Zhu, Y., Qiu, W., Liu, Y.J., Cheng, G., Liu, Z.J., and Ouyang, S. (2017). Structural and functional analyses of human DDX41 DEAD domain. Protein Cell 8, 72-76.

Laskowski, R.A. (2007). Enhancing the functional annotation of PDB structures in PDBsum using key figures extracted from the literature. Bioinformatics 23, 1824-1827.

Otwinowski, Z., and Minor, W. (1997). [20] Processing of X-ray diffraction data collected in oscillation mode. Methods Enzymol 276, 307-326.

Wang, Q., Guan, Z., Pei, K., Wang, J., Liu, Z., Yin, P., Peng, D., and Zou, T. (2018). Structural basis of the arbitrium peptide-AimR communication system in the phage lysis-lysogeny decision. Nat Microbiol.

Winn, M.D., Ballard, C.C., Cowtan, K.D., Dodson, E.J., Emsley, P., Evans, P.R., Keegan, R.M., Krissinel, E.B., Leslie, A.G., McCoy, A., et al. (2011). Overview of the CCP4 suite and current developments. Acta Crystallogr D Biol Crystallogr 67, 235-242.
